# A Comparative Analysis Between Vertical Rectus Abdominis Myocutaneous (VRAM) Flap and Transverse Rectus Abdominis Myocutaneous (TRAM) Flap as Options for Post-mastectomy Chest Wall Reconstruction

**DOI:** 10.7759/cureus.88321

**Published:** 2025-07-19

**Authors:** Antarip Bhattacharya, Dhritiman Maitra

**Affiliations:** 1 General Surgery, Medical College Kolkata, Kolkata, IND; 2 General Surgery, Newham University Hospital, Barts Health NHS Trust, London, GBR

**Keywords:** breast cancer surgery, breast reconstruction, chest wall reconstruction, oncoplastic surgery, pedicled flap reconstruction, tram flap, vram flap

## Abstract

Background

Oncoplastic breast reconstruction mitigates chest wall deformities following mastectomy or post-radiation therapy after lumpectomy. It is associated with lower morbidity, improved quality of life, and more natural aesthetic outcomes compared to traditional techniques. While free flaps yield excellent cosmetic results, their reliance on advanced microsurgical expertise poses challenges in resource-limited settings. In such contexts, pedicled flaps like the Vertical Rectus Abdominis Myocutaneous (VRAM) and Transverse Rectus Abdominis Myocutaneous (TRAM) flaps provide safe, reliable alternatives.

Patients and methods

This retrospective observational study analysed 45 patients who underwent post-mastectomy chest wall reconstruction at Medical College Hospital, Kolkata, between October 2020 and March 2024. Twenty-three patients received VRAM flaps (Group A), and twenty-two received TRAM flaps (Group B). The groups were compared regarding the timing of adjuvant therapy initiation, postoperative complications, donor-site morbidity, recurrence rates, and adequacy of tissue coverage.

Results

Adjuvant therapy was initiated in 21/23 (91.3%) of Group A (95% CI: 73.2% to 97.6%) compared to 11/22 (50.0%) of Group B (95% CI: 30.7% to 69.3%) (p=0.02). Flap necrosis occurred in 2/23 (8.7%) of Group A (95% CI: 2.4% to 26.8%) vs. 11/22 (50.0%) of Group B (95% CI: 30.7% to 69.3%) (p=0.01). Donor-site skin necrosis was absent in Group A 0/23 (0.0%) (95% CI: 0.0% to 14.3%) but occurred in 7/22 (31.8%) of Group B (95% CI: 16.4% to 52.7%) (p=0.02). Umbilical necrosis and activity limitation were only reported in Group B, each in 2/22 (9.1%) of patients (95% CI for both: 2.5% to 27.8%). Both groups achieved complete defect coverage and had no recurrences at six months.

Conclusion

VRAM flaps offer superior outcomes in terms of reduced postoperative complications and earlier initiation of adjuvant therapy, making them preferable in resource-constrained environments. However, longer follow-up and larger cohorts are needed to validate these findings.

## Introduction

Oncoplastic reconstruction, introduced in the 1980s, plays a pivotal role in minimising post-mastectomy deformities. It improves cosmetic outcomes and preserves function in comparison to conventional reconstruction [1]. Challenges such as tissue fibrosis and compromised vascularity from radiotherapy necessitate robust reconstructive strategies [2].

Local muscle flaps, including the pectoralis major, latissimus dorsi, serratus anterior, rectus abdominis, and occasionally the omentum, are often employed [3]. Free flaps like the Deep Inferior Epigastric Artery Perforator (DIEP) flap offer excellent results but require advanced microsurgical skills and resources [4].

Pedicled flaps such as Vertical Rectus Abdominis Myocutaneous (VRAM) and Transverse Rectus Abdominis Myocutaneous (TRAM) provide viable alternatives in settings with limited resources. The Rectus Abdominis Myocutaneous (RAM) flap, first described by Robbins in 1979 (vertical paddle) and modified by Hartrampf in 1982 (transverse paddle), has become a standard in reconstruction [5].

The perfusion of TRAM flaps has been mapped into four zones, with perfusion decreasing from Zone I (pedicle side) to Zone IV (contralateral lateral) [6]. The rectus abdominis muscle, with dual blood supply from the deep superior and inferior epigastric arteries, allows for reliable pedicled transfer either superiorly or inferiorly [7].

The VRAM flap, superiorly based on the deep superior epigastric artery, benefits from a more direct muscular blood supply, particularly above the arcuate line. This anatomical advantage may account for its reduced complication rate compared to TRAM.

## Materials and methods

Patient characteristics

The study population comprised 45 female patients aged between 40 and 55 years, all of whom underwent post-mastectomy chest wall reconstruction using either a VRAM (n=23) or TRAM (n=22) flap. All patients had a body mass index (BMI) between 20 and 26 and were non-smokers. Among them, 21 were pre-menopausal and 24 were post-menopausal. Eleven patients (24.4%) had relevant co-morbidities, including type 2 diabetes mellitus and hypertension.

All patients with locally advanced breast cancer (LABC) had received neoadjuvant chemotherapy (NACT), but the disease was non-responsive, necessitating surgical excision. The remaining patients had extensive phyllodes tumours, many of which were fungating, ulcerated, or secondarily infected. One patient had maggot infestation and features of systemic sepsis at presentation. These clinical presentations led to large and complex chest wall defects following mastectomy, necessitating immediate and reliable flap coverage.

**Figure 1:**
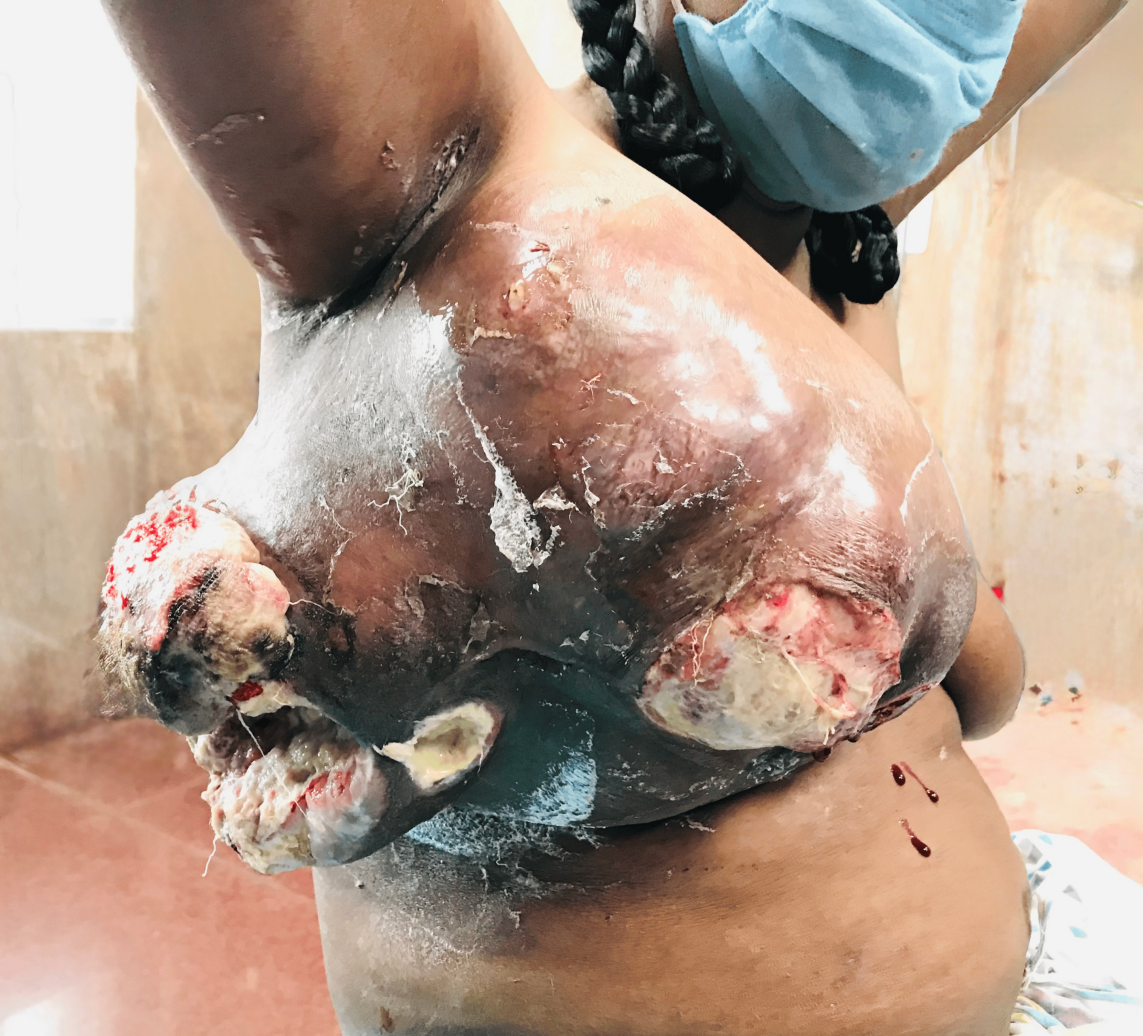
Large fungating phyllodes tumour of a patient

This demographic and clinical homogeneity ensured that both groups were comparable in terms of baseline risk factors, allowing a focused evaluation of flap outcomes in a real-world, resource-limited setting. The clinical presentation of one patient with a large, fungating and secondarily infected phyllodes tumour is depicted in Figure 1.

Study design

This was a retrospective observational study conducted at the Department of General Surgery, Medical College Kolkata, a tertiary care multidisciplinary hospital. The study period extended from October 2020 to March 2024. The study population comprised all patients who underwent chest wall reconstruction following mastectomy using either VRAM or TRAM flaps during the specified period. Records of a total of 45 patients were reviewed. Twenty-three patients underwent reconstruction with VRAM flaps (Group A), and twenty-two with TRAM flaps (Group B). Patient outcomes were evaluated in terms of postoperative complications, donor-site morbidity, time to initiation of adjuvant therapy, adequacy of tissue coverage, and short-term recurrence.

Surgical technique

All surgeries were performed by a single surgeon trained in oncoplastic breast surgery, ensuring uniformity in technique and minimising inter-operator variability. For LABC, modified radical mastectomy (MRM) was performed and for phyllodes tumours, radical mastectomy without axillary clearance was undertaken. Defects were typically large due to tumour bulk, secondary infection, bleeding, and in one case, maggot infestation with sepsis.

**Figure 2:**
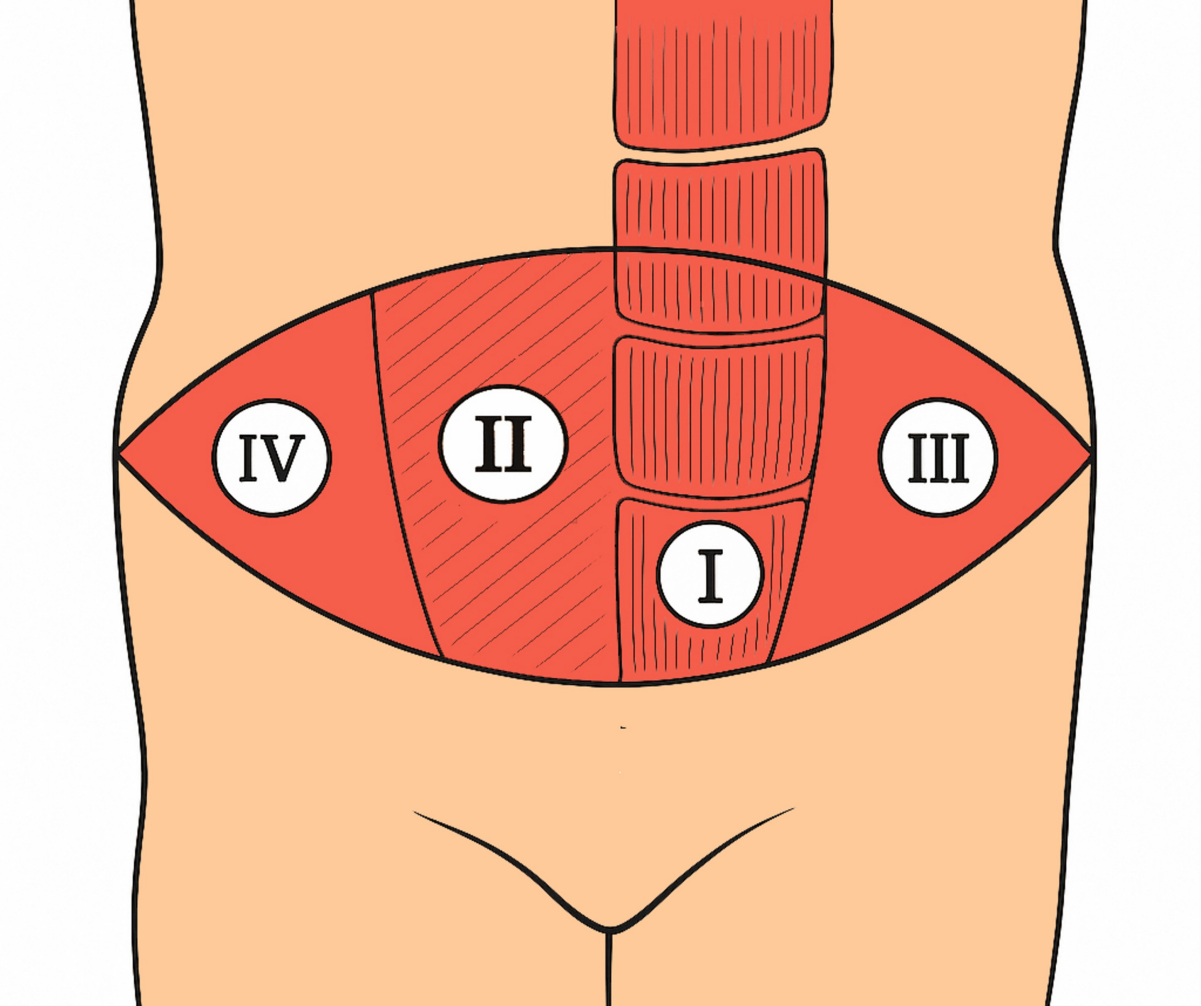
Schematic representation of the Hartrampf’s zones of perfusion Zone I (central) demonstrates optimal perfusion, followed by Zones II, III, and IV with progressively reduced vascularity. Schematic illustration by Antarip Bhattacharya

The TRAM flap was harvested using a transverse skin paddle based on the rectus abdominis muscle. The dominant vascular pedicle, typically the deep inferior epigastric artery, was preserved during dissection. Care was taken to avoid injury to perforators and to ensure adequate muscle and skin coverage for defect reconstruction. The flap was then rotated into the defect area and inset with minimal tension. Zone IV was discarded in all cases due to poor perfusion. The donor site was closed primarily with or without mesh reinforcement depending on the integrity of the abdominal wall. Subcutaneous layers were closed using Vicryl and skin was approximated using Monocryl, Ethilon or staples depending on wound tension and location. Flap design and dissection were guided by the established vascular zones of the rectus abdominis muscle, as described by Hartrampf, with Zone I having the most reliable perfusion and Zone IV the least [5]. These zones and their relevance to flap planning are illustrated in Figure 2.

**Figure 3:**
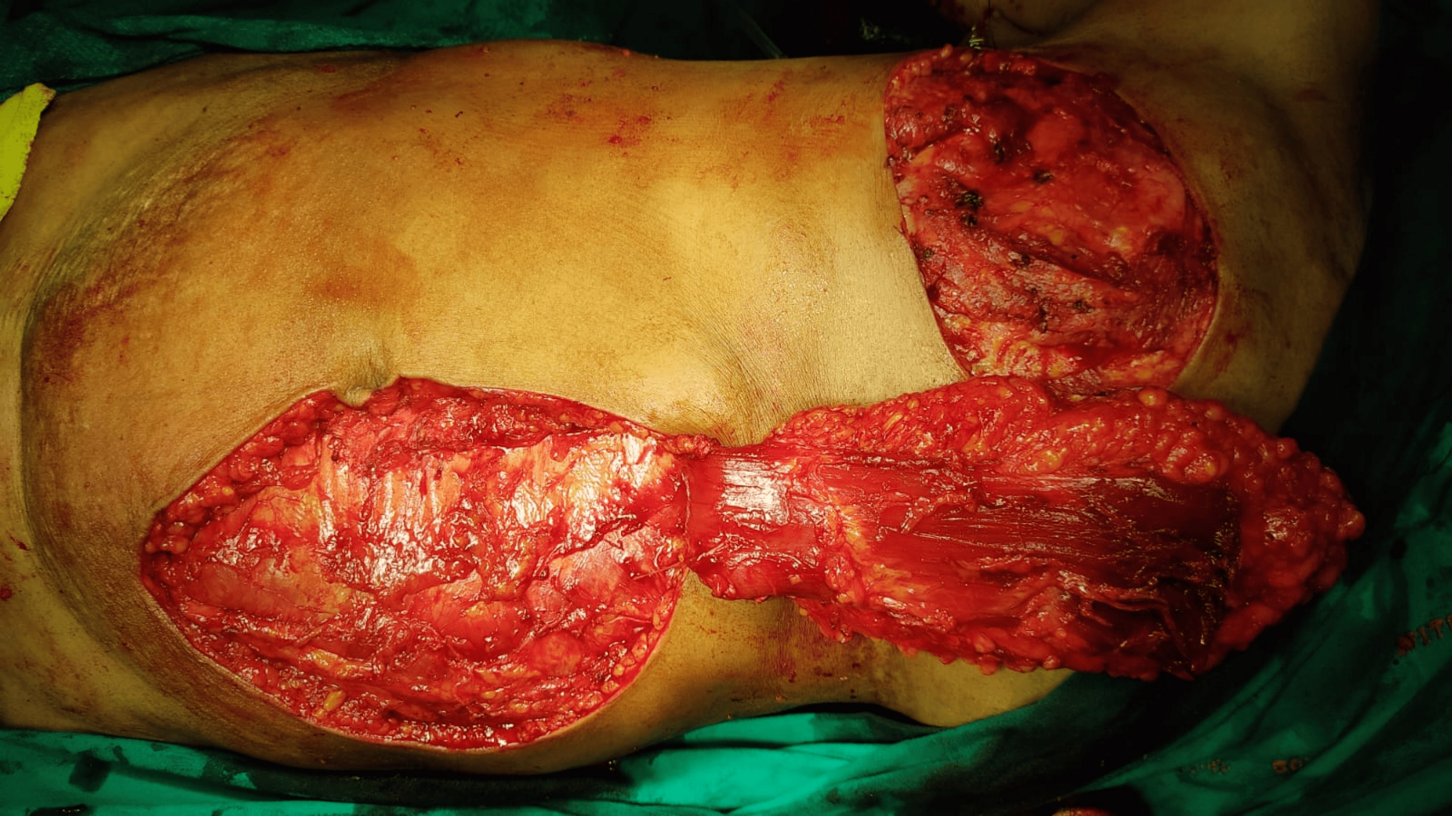
Harvesting the VRAM flap VRAM: Vertical Rectus Abdominis Myocutaneous

The surgical technique for harvesting the VRAM flap involved elevation of the rectus abdominis muscle based on the deep superior epigastric artery, ensuring adequate skin paddle perfusion and vascular integrity. The flap was rotated to the chest wall defect while preserving the muscle pedicle. The closure techniques were the same as in TRAM flap. A representative image of this procedure is shown in Figure 3.

Ethical considerations

This study was conducted in accordance with institutional guidelines for retrospective analyses of anonymised data. As the study involved no direct patient contact or intervention, and all data were de-identified, it was exempt from formal ethics committee review. All patients consented for medical photography and its anonymised use for teaching and research during the procedure and on follow-up.

Statistical analysis

Data were compiled and analysed using standard statistical software (SPSS version 25.0, IBM Corp., Armonk, NY, USA). Categorical variables were compared using two-tailed Chi-square test or Fisher’s exact test, as appropriate. Continuous variables were expressed as mean or median with standard deviation or range, though not detailed due to sample size. Confidence intervals were calculated at a 95% level for proportions and reported where appropriate. A p-value less than 0.05 was considered statistically significant.

## Results

Recurrence

No patients in either group had recurrence at the six-month follow-up.

Tissue coverage

**Figure 4:**
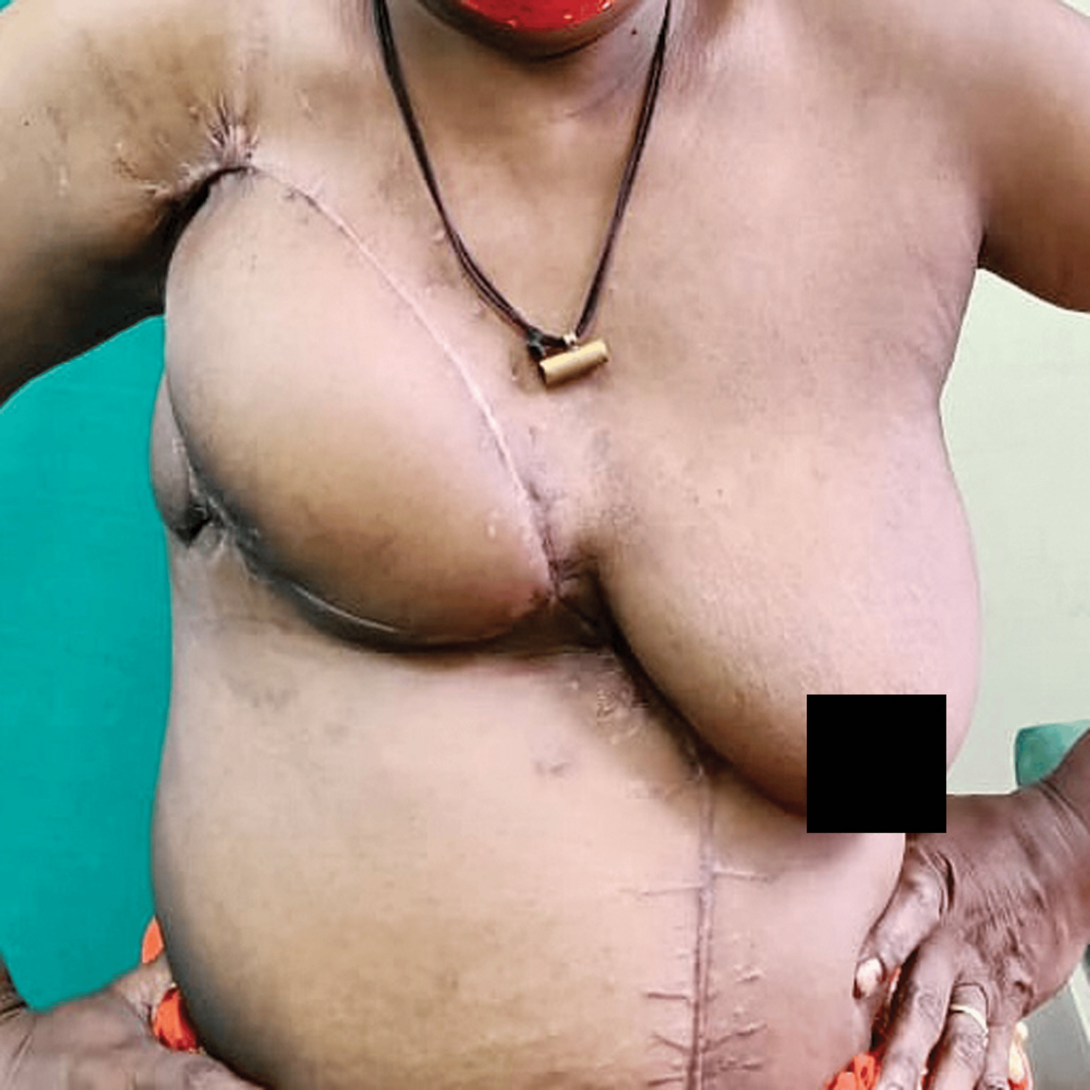
Postoperative result of VRAM flap showing complete tissue coverage of the chest wall with satisfactory wound healing, including the donor site VRAM: Vertical Rectus Abdominis Myocutaneous

Complete coverage of the chest wall defect was achieved in all patients (100%) in both groups, as demonstrated in Figure 4.

Initiation of adjuvant therapy

Adjuvant therapy could be initiated in 21 out of 23 (91.3%) patients in Group A (95% CI: 73.2% to 97.6%), compared to 11 out of 22 (50.0%) in Group B (95% CI: 30.7% to 69.3%) (p=0.02).

Flap necrosis

**Figure 5:**
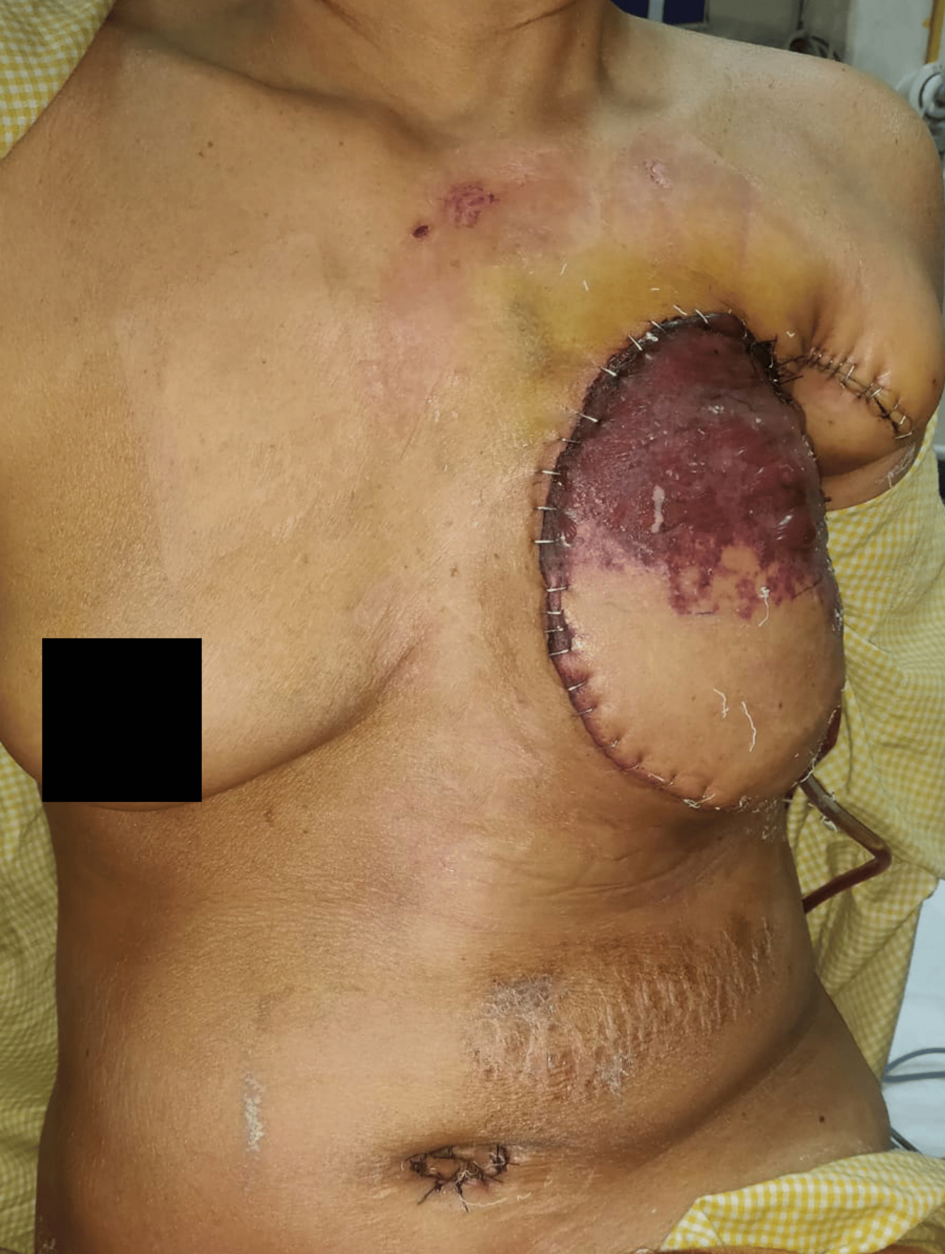
Early postoperative image of TRAM flap showing flap necrosis over the reconstructed chest wall, with dusky discolouration and loss of vascularity in the central portion of the flap TRAM: Transverse Rectus Abdominis Myocutaneous

Flap necrosis was observed in two patients (8.7%) in Group A (95% CI: 2.4% to 26.8%), compared to 11 patients (50.0%) in Group B (95% CI: 30.7% to 69.3%) (p=0.01). This is demonstrated in Figure 5.

Donor-site skin necrosis

**Figure 6:**
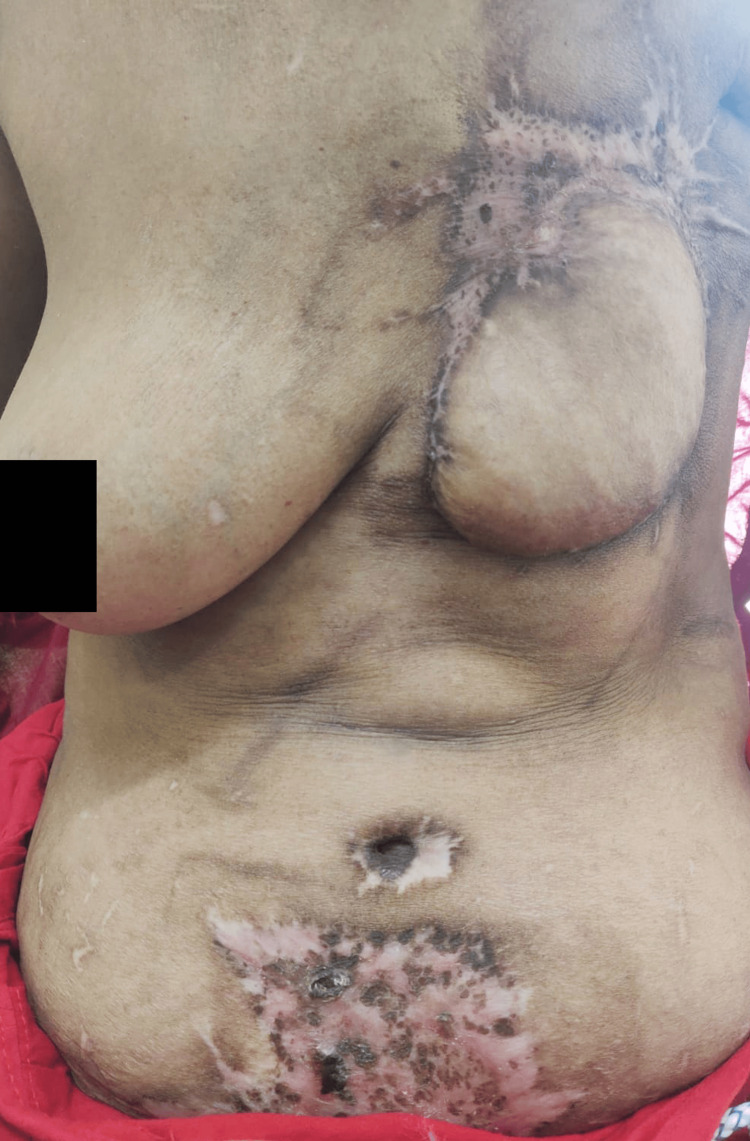
Donor-site skin of TRAM flap after debridement and split-thickness skin grafting, showing areas of graft take interspersed with scab formation TRAM: Transverse Rectus Abdominis Myocutaneous

Donor-site skin necrosis was not reported in Group A (95% CI: 0.0% to 14.3%), whereas it occurred in seven patients (31.8%) in Group B (95% CI: 16.4% to 52.7%) (p=0.02). This is demonstrated in Figure 6.

Umbilical necrosis

There were no cases of umbilical necrosis in Group A (95% CI: 0.0% to 14.3%). Two patients (9.1%) in Group B developed umbilical necrosis (95% CI: 2.5% to 27.8%) (p=0.3).

Stiffness and limitation of daily activity

No patients in Group A reported postoperative stiffness or activity limitation (95% CI: 0.0% to 14.3%). Two patients (9.1%) in Group B experienced activity limitation (95% CI: 2.5% to 27.8%) (p=0.3).

A detailed comparison of outcomes is presented in Table 1.

**Table 1:** Comparison of Outcomes Between Groups Statistically significant p-values (p < 0.05) are highlighted in bold.

Parameter	Group A (n = 23)	Group B (n = 22)	p-value
Recurrence at 6 Months	0 (0%)	0 (0%)	–
Tissue Coverage	23 (100%)	22 (100%)	–
Initiation of Adjuvant Therapy	21 (91.3%)	11 (50.0%)	0.02
Flap Necrosis	2 (8.7%)	11 (50.0%)	0.01
Donor-Site Skin Necrosis	0 (0%)	7 (31.8%)	0.02
Umbilical Necrosis	0 (0%)	2 (9.1%)	0.3
Stiffness/Activity Limitation	0/23 (0%)	2/22 (9.1%)	0.3

## Discussion

The present study compares the outcomes of VRAM and TRAM flaps for post-mastectomy chest wall reconstruction. The results demonstrate that VRAM flaps are associated with significantly fewer postoperative complications and allow earlier initiation of adjuvant therapy compared to TRAM flaps. These findings align with the existing literature, which highlights the superior vascular reliability of the VRAM flap, attributed to its robust blood supply from the deep superior epigastric artery [8].

Flap necrosis remains a critical concern in reconstructive surgery. In this study, the incidence of flap necrosis was notably lower in the VRAM group, corroborating the findings of Kotti, who reported no flap-related complications in VRAM reconstructions, while TRAM flaps were associated with partial necrosis rates as high as 35% when classical designs were used [8]. The revised vascular zoning and safer skin paddle designs proposed by Kotti and colleagues may explain these improved outcomes with VRAM flaps. Incorporating such design considerations into clinical practice can significantly reduce the risk of ischemic complications.

The earlier initiation of adjuvant therapy observed in the VRAM group (21/23, 91.3%) compared to the TRAM group (11/22, 50.0%) is of paramount importance in oncological management. Delays in initiating chemotherapy or radiotherapy can adversely impact long-term survival outcomes. Mir et al. similarly observed that immediate breast reconstruction using VRAM or TRAM flaps in patients with Stage IIb and III breast cancer did not result in significant delays in adjuvant therapy, although flap-related complications did contribute to minor delays in some cases [9]. Our study further supports the safety and oncological appropriateness of immediate reconstruction using VRAM flaps, even in patients requiring early postoperative systemic therapy.

Donor-site morbidity is another crucial consideration. This study found no cases of donor-site necrosis or umbilical complications in the VRAM group, a finding consistent with Dragu et al., who emphasised that the VRAM flap is particularly suitable for patients with previous abdominal surgeries or vertical scars, due to the preserved vascular territories in the periumbilical region [10]. Their findings suggest that the VRAM flap remains a viable option even in complex cases where other reconstructive methods may be contraindicated. Furthermore, in a large retrospective study by Joel et al., VRAM flaps were found to be effective in achieving adequate coverage for large thoracic wall defects in patients with locally advanced and metastatic breast cancer, with acceptable complication rates and a mean survival of 25.5 months [11].

From a functional standpoint, patients in the VRAM group reported no postoperative limitations in daily activities, underscoring the flap's advantages in preserving abdominal wall integrity and minimizing morbidity. This observation is in line with the conclusions drawn by Mir et al., who noted positive psychological and functional outcomes in patients undergoing immediate VRAM reconstruction [9].

In terms of long-term outcomes, Blondeel et al. emphasised that preservation of abdominal wall function and minimisation of donor-site morbidity remain critical determinants of patient satisfaction in breast reconstruction [12]. Our findings support this notion, as patients in the VRAM group experienced fewer functional limitations compared to the TRAM group.

Limitations

The primary limitation of this study is its retrospective design and relatively small sample size. This introduces the possibility of selection bias, as patient allocation and data collection were not prospectively controlled. Additionally, the single-surgeon, single-centre nature of the study limits its reproducibility and generalisability, despite providing procedural consistency. Additionally, the short follow-up period precludes the assessment of long-term oncological outcomes and late complications such as incisional hernias or bulging. While we acknowledge that superior reconstructive techniques exist, including microsurgical free flaps and composite flaps with better aesthetic and functional outcomes, these options were not feasible in our context due to a lack of infrastructure and financial constraints among our predominantly low-income patient population. Given these limitations, our use of pedicled VRAM and TRAM flaps prioritised infection control, tumour removal, timely closure and early initiation of adjuvant therapy. Furthermore, symmetrisation was not routinely performed in this cohort, as the primary objective was functional chest wall coverage rather than aesthetic breast reconstruction. Future prospective studies with larger patient cohorts and extended follow-up periods are needed to validate these findings and explore patient-reported quality-of-life metrics.

## Conclusions

This retrospective analysis suggests that VRAM flaps are associated with fewer postoperative complications and allow earlier initiation of adjuvant therapy compared to TRAM flaps, while achieving satisfactory oncologic and reconstructive outcomes. These findings highlight the VRAM flap as a preferred reconstructive option in resource-constrained settings due to its lower complication rates, and better functional and quality-of-life outcomes.

Future prospective studies with larger cohorts and longer follow-up are needed to validate these findings. Research should also assess patient-reported quality of life and cost-effectiveness to further guide reconstructive decision-making in various healthcare settings.
